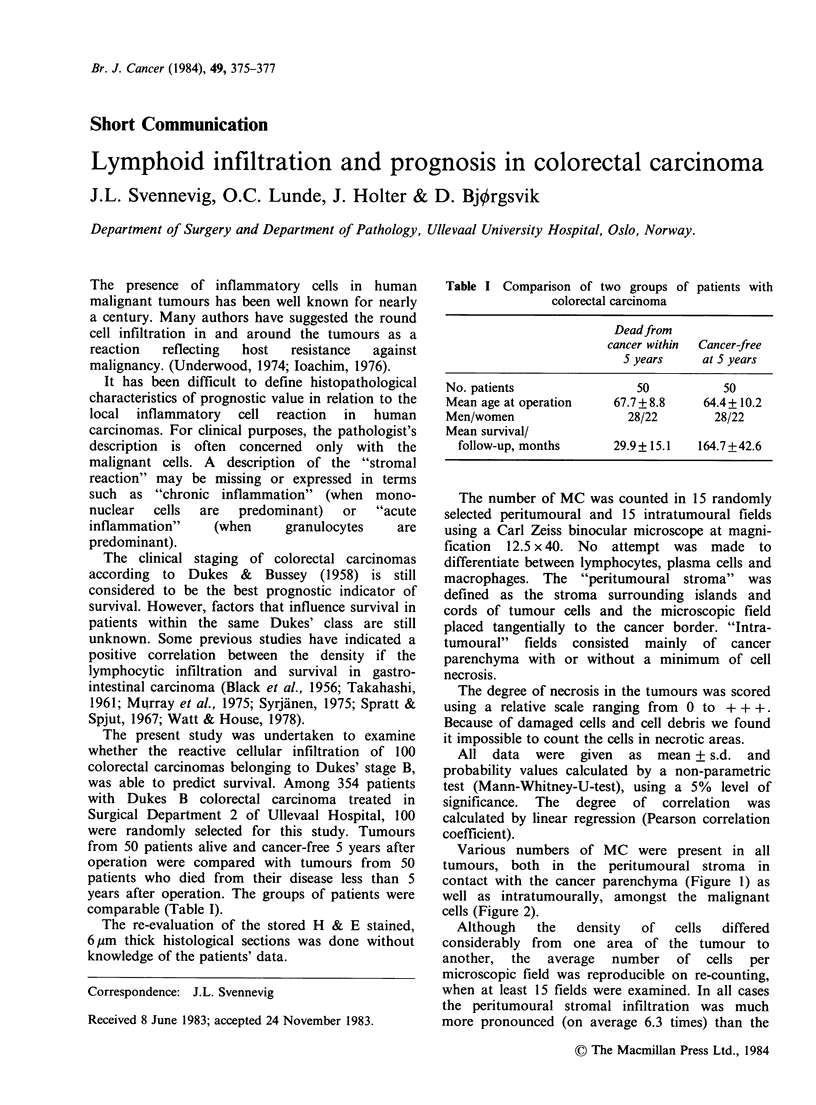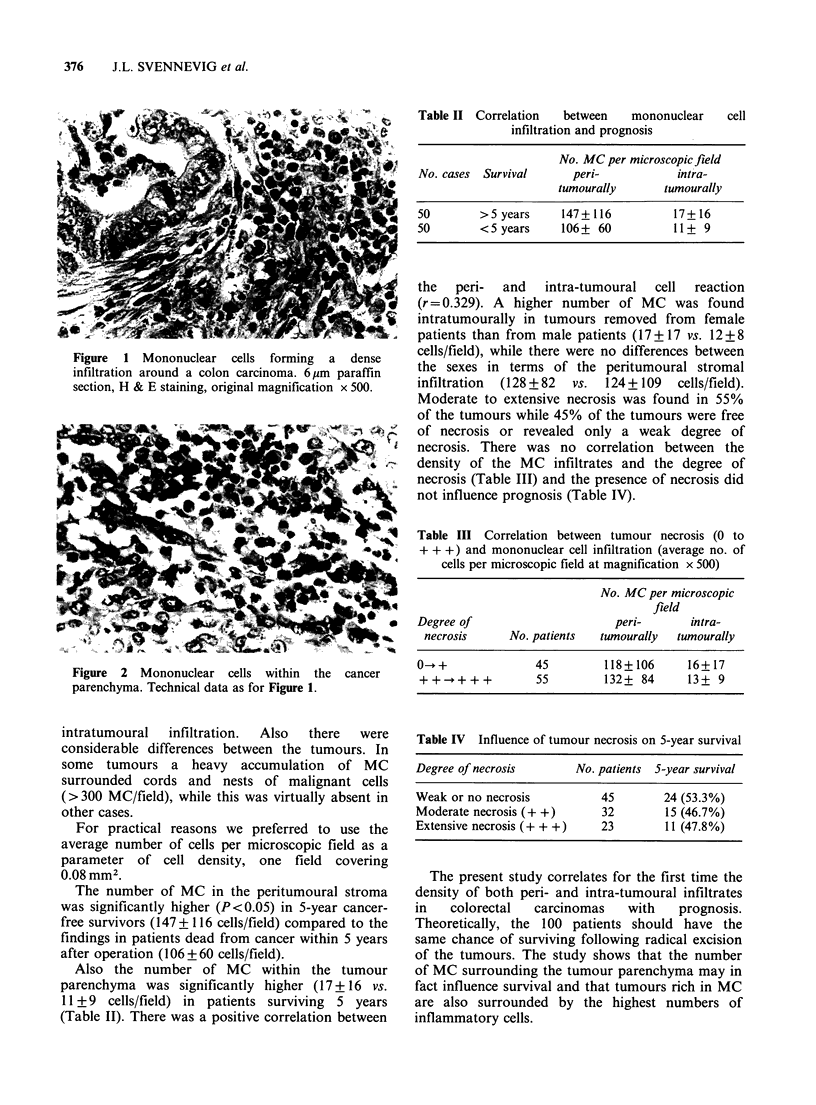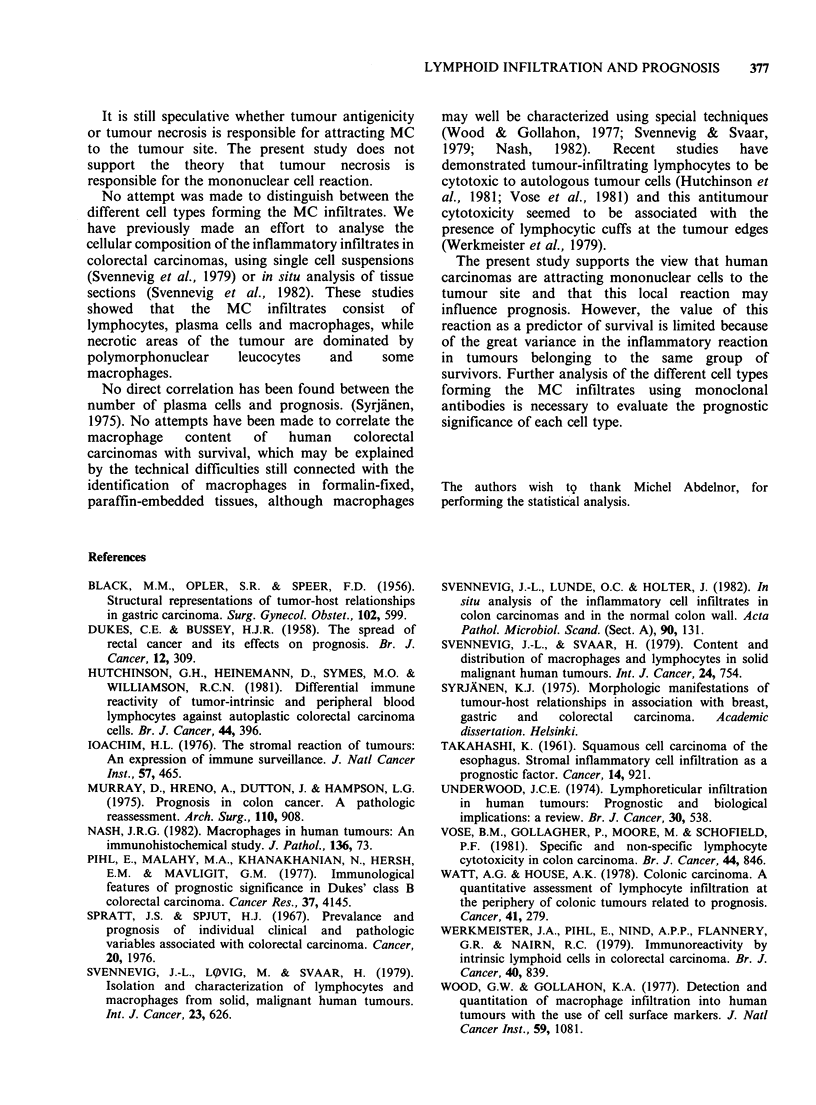# Lymphoid infiltration and prognosis in colorectal carcinoma.

**DOI:** 10.1038/bjc.1984.60

**Published:** 1984-03

**Authors:** J. L. Svennevig, O. C. Lunde, J. Holter, D. Bjørgsvik

## Abstract

**Images:**


*Br. J. Cancer* (1984), **49**, 375–377

### Short Communication

### Lymphoid infiltration and prognosis in colorectal carcinoma

J.L. Svennevig, O.C. Lunde, J. Holter & D. Bjørgsvik

*Department of Surgery and Department of Pathology, Ullevaal University Hospital, Oslo, Norway.*

The presence of inflammatory cells in human malignant tumours has been well known for nearly a century. Many authors have suggested the round cell infiltration in and around the tumours as a reaction reflecting host resistance against malignancy. (Underwood, 1974; Ioachim, 1976).

It has been difficult to define histopathological characteristics of prognostic value in relation to the local inflammatory cell reaction in human carcinomas. For clinical purposes, the pathologist's description is often concerned only with the malignant cells. A description of the "stromal reaction" may be missing or expressed in terms such as "chronic inflammation" (when mononuclear cells are predominant) or "acute inflammation" (when granulocytes are predominant).

The clinical staging of colorectal carcinomas according to Dukes & Bussey (1958) is still considered to be the best prognostic indicator of survival. However, factors that influence survival in patients within the same Dukes' class are still unknown. Some previous studies have indicated a positive correlation between the density if the lymphocytic infiltration and survival in gastrointestinal carcinoma (Black *et al.*, 1956; Takahashi, 1961; Murray *et al.*, 1975; Syrjänen, 1975; Spratt & Spjut, 1967; Watt & House, 1978).

The present study was undertaken to examine whether the reactive cellular infiltration of 100 colorectal carcinomas belonging to Dukes' stage B, was able to predict survival. Among 354 patients with Dukes B colorectal carcinoma treated in Surgical Department 2 of Ullevaal Hospital, 100 were randomly selected for this study. Tumours from 50 patients alive and cancer-free 5 years after operation were compared with tumours from 50 patients who died from their disease less than 5 years after operation. The groups of patients were comparable (Table I).

The re-evaluation of the stored H & E stained, 6 μm thick histological sections was done without knowledge of the patients' data.

Correspondence: J.L. Svennevig

Received 8 June 1983; accepted 24 November 1983.

**Table I** Comparison of two groups of patients with colorectal carcinoma

| | *Dead from cancer within 5 years* | *Cancer-free at 5 years* |
|---|---|---|
| No. patients | 50 | 50 |
| Mean age at operation | 67.7±8.8 | 64.4±10.2 |
| Men/women | 28/22 | 28/22 |
| Mean survival/ follow-up, months | 29.9±15.1 | 164.7±42.6 |

The number of MC was counted in 15 randomly selected peritumoural and 15 intratumoural fields using a Carl Zeiss binocular microscope at magnification 12.5×40. No attempt was made to differentiate between lymphocytes, plasma cells and macrophages. The "peritumoural stroma" was defined as the stroma surrounding islands and cords of tumour cells and the microscopic field placed tangentially to the cancer border. "Intratumoural" fields consisted mainly of cancer parenchyma with or without a minimum of cell necrosis.

The degree of necrosis in the tumours was scored using a relative scale ranging from 0 to +++. Because of damaged cells and cell debris we found it impossible to count the cells in necrotic areas.

All data were given as mean ± s.d. and probability values calculated by a non-parametric test (Mann-Whitney-U-test), using a 5% level of significance. The degree of correlation was calculated by linear regression (Pearson correlation coefficient).

Various numbers of MC were present in all tumours, both in the peritumoural stroma in contact with the cancer parenchyma (Figure 1) as well as intratumourally, amongst the malignant cells (Figure 2).

Although the density of cells differed considerably from one area of the tumour to another, the average number of cells per microscopic field was reproducible on re-counting, when at least 15 fields were examined. In all cases the peritumoural stromal infiltration was much more pronounced (on average 6.3 times) than the

© The Macmillan Press Ltd., 1984

**Figure 1** Mononuclear cells forming a dense infiltration around a colon carcinoma. 6 μm paraffin section, H & E staining, original magnification × 500.

**Figure 2** Mononuclear cells within the cancer parenchyma. Technical data as for **Figure 1**.

intratumoural infiltration. Also there were considerable differences between the tumours. In some tumours a heavy accumulation of MC surrounded cords and nests of malignant cells (>300 MC/field), while this was virtually absent in other cases.

For practical reasons we preferred to use the average number of cells per microscopic field as a parameter of cell density, one field covering 0.08 $mm^2$.

The number of MC in the peritumoural stroma was significantly higher ($P<0.05$) in 5-year cancer-free survivors (147 ± 116 cells/field) compared to the findings in patients dead from cancer within 5 years after operation (106 ± 60 cells/field).

Also the number of MC within the tumour parenchyma was significantly higher (17 ± 16 *vs.* 11 ± 9 cells/field) in patients surviving 5 years (Table II). There was a positive correlation between

**Table II** Correlation between mononuclear cell infiltration and prognosis

| | | *No. MC per microscopic field* | |
|---|---|---|---|
| *No. cases* | *Survival* | *peri-tumourally* | *intra-tumourally* |
| 50 | >5 years | 147 ± 116 | 17 ± 16 |
| 50 | <5 years | 106 ± 60 | 11 ± 9 |

the peri- and intra-tumoural cell reaction ($r=0.329$). A higher number of MC was found intratumourally in tumours removed from female patients than from male patients (17 ± 17 *vs.* 12 ± 8 cells/field), while there were no differences between the sexes in terms of the peritumoural stromal infiltration (128 ± 82 *vs.* 124 ± 109 cells/field). Moderate to extensive necrosis was found in 55% of the tumours while 45% of the tumours were free of necrosis or revealed only a weak degree of necrosis. There was no correlation between the density of the MC infiltrates and the degree of necrosis (Table III) and the presence of necrosis did not influence prognosis (Table IV).

**Table III** Correlation between tumour necrosis (0 to + + +) and mononuclear cell infiltration (average no. of cells per microscopic field at magnification × 500)

| | | *No. MC per microscopic field* | |
|---|---|---|---|
| *Degree of necrosis* | *No. patients* | *peri-tumourally* | *intra-tumourally* |
| 0 → + | 45 | 118 ± 106 | 16 ± 17 |
| + + → + + + | 55 | 132 ± 84 | 13 ± 9 |

**Table IV** Influence of tumour necrosis on 5-year survival

| *Degree of necrosis* | *No. patients* | *5-year survival* |
|---|---|---|
| Weak or no necrosis | 45 | 24 (53.3%) |
| Moderate necrosis (+ +) | 32 | 15 (46.7%) |
| Extensive necrosis (+ + +) | 23 | 11 (47.8%) |

The present study correlates for the first time the density of both peri- and intra-tumoural infiltrates in colorectal carcinomas with prognosis. Theoretically, the 100 patients should have the same chance of surviving following radical excision of the tumours. The study shows that the number of MC surrounding the tumour parenchyma may in fact influence survival and that tumours rich in MC are also surrounded by the highest numbers of inflammatory cells.

It is still speculative whether tumour antigenicity or tumour necrosis is responsible for attracting MC to the tumour site. The present study does not support the theory that tumour necrosis is responsible for the mononuclear cell reaction.

No attempt was made to distinguish between the different cell types forming the MC infiltrates. We have previously made an effort to analyse the cellular composition of the inflammatory infiltrates in colorectal carcinomas, using single cell suspensions (Svennevig *et al.*, 1979) or *in situ* analysis of tissue sections (Svennevig *et al.*, 1982). These studies showed that the MC infiltrates consist of lymphocytes, plasma cells and macrophages, while necrotic areas of the tumour are dominated by polymorphonuclear leucocytes and some macrophages.

No direct correlation has been found between the number of plasma cells and prognosis. (Syrjänen, 1975). No attempts have been made to correlate the macrophage content of human colorectal carcinomas with survival, which may be explained by the technical difficulties still connected with the identification of macrophages in formalin-fixed, paraffin-embedded tissues, although macrophages may well be characterized using special techniques (Wood & Gollahon, 1977; Svennevig & Svaar, 1979; Nash, 1982). Recent studies have demonstrated tumour-infiltrating lymphocytes to be cytotoxic to autologous tumour cells (Hutchinson *et al.*, 1981; Vose *et al.*, 1981) and this antitumour cytotoxicity seemed to be associated with the presence of lymphocytic cuffs at the tumour edges (Werkmeister *et al.*, 1979).

The present study supports the view that human carcinomas are attracting mononuclear cells to the tumour site and that this local reaction may influence prognosis. However, the value of this reaction as a predictor of survival is limited because of the great variance in the inflammatory reaction in tumours belonging to the same group of survivors. Further analysis of the different cell types forming the MC infiltrates using monoclonal antibodies is necessary to evaluate the prognostic significance of each cell type.

The authors wish to thank Michel Abdelnor, for performing the statistical analysis.

### References

BLACK, M.M., OPLER, S.R. & SPEER, F.D. (1956). Structural representations of tumor-host relationships in gastric carcinoma. *Surg. Gynecol. Obstet.*, **102**, 599.

DUKES, C.E. & BUSSEY, H.J.R. (1958). The spread of rectal cancer and its effects on prognosis. *Br. J. Cancer*, **12**, 309.

HUTCHINSON, G.H., HEINEMANN, D., SYMES, M.O. & WILLIAMSON, R.C.N. (1981). Differential immune reactivity of tumor-intrinsic and peripheral blood lymphocytes against autoplastic colorectal carcinoma cells. *Br. J. Cancer*, **44**, 396.

IOACHIM, H.L. (1976). The stromal reaction of tumours: An expression of immune surveillance. *J. Natl Cancer Inst.*, **57**, 465.

MURRAY, D., HRENO, A., DUTTON, J. & HAMPSON, L.G. (1975). Prognosis in colon cancer. A pathologic reassessment. *Arch. Surg.*, **110**, 908.

NASH, J.R.G. (1982). Macrophages in human tumours: An immunohistochemical study. *J. Pathol.*, **136**, 73.

PIHL, E., MALAHY, M.A., KHANAKHANIAN, N., HERSH, E.M. & MAVLIGIT, G.M. (1977). Immunological features of prognostic significance in Dukes' class B colorectal carcinoma. *Cancer Res.*, **37**, 4145.

SPRATT, J.S. & SPJUT, H.J. (1967). Prevalance and prognosis of individual clinical and pathologic variables associated with colorectal carcinoma. *Cancer*, **20**, 1976.

SVENNEVIG, J.-L., LØVIG, M. & SVAAR, H. (1979). Isolation and characterization of lymphocytes and macrophages from solid, malignant human tumours. *Int. J. Cancer*, **23**, 626.

SVENNEVIG, J.-L., LUNDE, O.C. & HOLTER, J. (1982). *In situ* analysis of the inflammatory cell infiltrates in colon carcinomas and in the normal colon wall. *Acta Pathol. Microbiol. Scand.* (Sect. A), **90**, 131.

SVENNEVIG, J.-L., & SVAAR, H. (1979). Content and distribution of macrophages and lymphocytes in solid malignant human tumours. *Int. J. Cancer*, **24**, 754.

SYRJÄNEN, K.J. (1975). Morphologic manifestations of tumour-host relationships in association with breast, gastric and colorectal carcinoma. *Academic dissertation. Helsinki.*

TAKAHASHI, K. (1961). Squamous cell carcinoma of the esophagus. Stromal inflammatory cell infiltration as a prognostic factor. *Cancer*, **14**, 921.

UNDERWOOD, J.C.E. (1974). Lymphoreticular infiltration in human tumours: Prognostic and biological implications: a review. *Br. J. Cancer*, **30**, 538.

VOSE, B.M., GOLLAGHER, P., MOORE, M. & SCHOFIELD, P.F. (1981). Specific and non-specific lymphocyte cytotoxicity in colon carcinoma. *Br. J. Cancer*, **44**, 846.

WATT, A.G. & HOUSE, A.K. (1978). Colonic carcinoma. A quantitative assessment of lymphocyte infiltration at the periphery of colonic tumours related to prognosis. *Cancer*, **41**, 279.

WERKMEISTER, J.A., PIHL, E., NIND, A.P.P., FLANNERY, G.R. & NAIRN, R.C. (1979). Immunoreactivity by intrinsic lymphoid cells in colorectal carcinoma. *Br. J. Cancer*, **40**, 839.

WOOD, G.W. & GOLLAHON, K.A. (1977). Detection and quantitation of macrophage infiltration into human tumours with the use of cell surface markers. *J. Natl Cancer Inst.*, **59**, 1081.